# Preparation and Characterization of Surface Photocatalytic Activity with NiO/TiO_2_ Nanocomposite Structure

**DOI:** 10.3390/ma8074273

**Published:** 2015-07-13

**Authors:** Jian-Zhi Chen, Tai-Hong Chen, Li-Wen Lai, Pei-Yu Li, Hua-Wen Liu, Yi-You Hong, Day-Shan Liu

**Affiliations:** 1 Institute of Electro-Optical and Materials Science, National Formosa University, Huwei, Yunlin 63201, Taiwan; E-Mails: jerry61310@gmail.com (J.-Z.C.); dsliu@ios.nfu.edu.tw (P.-Y.L.); dsliu@nfu.edu.tw (H.-W.L.); a0922639175@gmail.com (Y.-Y.H.); 2 Industrial Technology Research Institute South, Liujia Shiang, Tainan 734, Taiwan; E-Mails: thchen1208@itri.org.tw (T.-H.C.); lwlai@itri.org.tw (L.-W.L.)

**Keywords:** nanocomposite structure, NiO/TiO_2_ heterojunction, photocatalytic activity, inner electrical field, photogenerated electron-hole pair

## Abstract

This study achieved a nanocomposite structure of nickel oxide (NiO)/titanium dioxide (TiO_2_) heterojunction on a TiO_2_ film surface. The photocatalytic activity of this structure evaluated by decomposing methylene blue (MB) solution was strongly correlated to the conductive behavior of the NiO film. A *p*-type NiO film of high concentration in contact with the native *n*-type TiO_2_ film, which resulted in a strong inner electrical field to effectively separate the photogenerated electron-hole pairs, exhibited a much better photocatalytic activity than the controlled TiO_2_ film. In addition, the photocatalytic activity of the NiO/TiO_2_ nanocomposite structure was enhanced as the thickness of the *p*-NiO film decreased, which was beneficial for the migration of the photogenerated carriers to the structural surface.

## 1. Introduction

In recent decades, the photocatalytic degradation of organic and inorganic pollutants using oxide semiconductor-based materials had been extensively studied [1,2,3,4,5]. Among these materials, titanium dioxide (TiO_2_) has been the most popular candidate due to its chemical stability, optical transparency at visible wavelength, high refractive index, non-toxic nature, and low cost [6,7,8]. Engineers have demonstrated that the TiO_2_ material with the anatase structure has the best photocatalytic activity [9,10]. However, as the photocatalytic activity of the crystalline TiO_2_ with anatase structure is constrained by the absorption edge at the UV wavelength, the recombination of the photo-generated electron-hole pairs, and the specific surface area, researchers have sought to modify the surface property of the TiO_2_ material by introducing metallic or non-metallic elements as well as enriching its reaction area. For instance, incorporating nitrogen and carbon elements into TiO_2_ films produces efficient ions, resulting in the light absorption shift of the film to the visible-light region [11,12], whereas the elements Fe, Ag, Pt, and F are commonly used as doping atoms in the TiO_2_ film to inhibit the recombination of the carriers under light irradiation [13,14,15,16]. In addition, the photocatalytic activity of TiO_2_ film was effectively improved with the achievement of a nano-texture on the film’s surface, which was beneficial for increasing the adsorption of all reactants [16,17]. Currently, constructing a heterojunction between TiO_2_ and semiconductors or oxide materials with appropriate band gap and band position, such as CdS, Cu_2_O, ZnO, Bi_2_O_3_, SrIn_2_O_4_, RuO_2_, and NiO, has offered a promising alternative to effectively enhance the photocatalytic activity of TiO_2_ [18,19,20,21,22,23,24,25]. For instance, Uddin *et al.* [23,25] used an impregnation method to introduce a transition-metal oxide of ruthenium oxide (RuO_2_) which possessed a high work function of about 6.1 eV on the sol-gel processed TiO_2_ nanopowders. The resulting RuO_2_/TiO_2_ mesoporous heterojunction exhibited a significant enhancement in the photocatalytic activity due to the improved separation of photogenetrated electron-hole pairs resulting from the internal electric field. Furthermore, since the TiO_2_ material is a well-known *n*-type material, nickel oxide (NiO) which shows a *p*-type semiconducting nature with a high band gap of about 3.86 eV [26], it is also expected to be a promising candidate on contact to TiO_2_ since an additive pathway associated with the formation of an inner electrical field between the heterojunction which is favorable for the separation of the photo-generated electrons and holes to different semiconductors in the *p*-NiO/*n*-TiO_2_ heterojunction structure, can be achieved. Although a few groups have had success in preparing a NiO/TiO_2_ heterojunction and the improvements in the resulting photocatalytic activity as a mechanism of the *p-n* junction have been discussed briefly [20,27], the photocatalytic activity of the NiO/TiO_2_ heterojunction structure influenced by the conduction behavior and thickness of the NiO film has been rarely investigated.

In this work, we developed a simple method to achieve the NiO/TiO_2_ nanocomposite structure on a TiO_2_ film surface via a rapid thermal annealing (RTA) process. The photocatalytic activities of the annealed NiO/TiO_2_ nanocomposite structures correlating with the electrical properties and crystalline structures of the NiO films, were evaluated and discussed. Evidence of the separation of the photo-generated electron-hole pairs achieved from these NiO/TiO_2_ nanocomposite structures was determined from the on-off process of the photocurrent response. In addition, the thickness of the NiO film in the NiO/TiO_2_ nanocomposite structure was varied in order to understand the resulting photocatalytic activity influenced by the diffusion of the photogenerated carriers in the NiO film to the structural surface.

## 2. Material Preparation and Experimental Procedure

Hydro-oxygenated amorphous titanium dioxide (TiO_2_) films with a thickness of 200 nm were deposited onto silicon and quartz substrates after a 20 nm-thick nickel seed layered evaporation and a sequential oxidation at 550 °C for 1 min under oxygen ambient, using plasma enhanced chemical vapor deposition (PECVD) at a temperature of 200 °C. A titanium tetra-isopropoxide [Ti(OC_3_H_7_)_4_, TTIP] liquid source was utilized as the titanium precursor through a heated bubbling cylinder at 70 °C, with nitrogen as the carrier gas and intermixed with pure oxygen gas in a mix box. All the gas pipelines were heated to 100 °C to prevent condensation of the precursor and gas mixture. The deposition pressure, rf power, and gas flow rate of the TTIP/O_2_ gas mixture were controlled at 40 Pa, 100 W, and 120/20 sccm, respectively. To optimize the film’s photocatalytic activity, these amorphous TiO_2_ films deposited onto the seed layer were then annealed at 600 °C for 30 min under ambient oxygen to result in the film crystallization with the anatase phase (denoted as the controlled TiO_2_ film hereafter). The reason for the TiO_2_ film deposited onto the seed layer and the mechanism responsible for the controlled TiO_2_ film exhibiting a better photocatalytic activity than that of the TiO_2_ film directly deposited onto the substrate, has been discussed elsewhere [28]. A metallic Ni film with a thickness of 20 nm was then evaporated onto the controlled TiO_2_ film surface, and sequentially annealed at 450 °C, 550 °C and 650 °C, respectively, under oxygen ambient for 1 min to realize the surface with the NiO/TiO_2_ nanocomposite structure. Another set of 200 nm-thick Ni films directly deposited onto the silicon substrate were also prepared and treated using the same RTA process to investigate the evolution of the film’s electrical and crystalline properties. In addition, the thickness of the Ni film deposited on the controlled TiO_2_ film was varied from 10 to 50 nm to further optimize the photocatalytic activity achieved by the NiO/TiO_2_ nanocomposite structure.

The film thickness of the controlled TiO_2_ film, the pure NiO films, and the samples with the NiO/TiO_2_ nanocomposite structure were measured using a surface profile system (Dektak 6M, Veeco, New York, NY, USA). Surface roughness and morphologies were examined using atomic force microscopy (AFM, DI-3100, Veeco, New York, NY, USA) and a field emission scanning electron microscope (FE-SEM, JSM-6700F, JEOL, Tokyo, Japan) operated at 3 kV. The chemical bond states and optical transmittance of the TiO_2_ films with and without the surface heterojunction structure were examined by Fourier transform infrared (FTIR) spectrometry (FT/IR-4100, JASCO, Halifax, NS, Canada) and a UV-Vis-NIR spectrophotometer (UVD 3500, Labomed, Inc., Los Angeles, CA, USA), respectively. The crystalline structure of these two samples and the pure NiO films annealed at various temperatures as well as the as-deposited Ni film were examined using a grazing incident X-ray diffractometer (GIXRD) at 30 kV and 30 mA using Cu*Kα_1_* radiation (D-500, Siemens, Munich, Germany). The electrical properties of the controlled TiO_2_ film and the Ni films treated by the RTA process were measured using the van der Pauw Hall measurements (HMS-5000, Ecopia Anyang, Korea) at room temperature. The photocatalytic activity of these TiO_2_ films with and without the surface heterojunction structure as well as the pure NiO film was evaluated from the decolorization of an aqueous methylene blue (MB) solution (20 mg·L^−1^ with a pH value approximately of 7.0 ± 0.1) at ambient temperature using a UV light irradiation (λ = 365 nm) at a constant intensity of 1 mW·cm^−2^. The photo-excited currents for the TiO_2_ films both with and without the surface NiO/TiO_2_ heterojunction, irradiated by a Xe lamp, were also conducted by a semiconductor parameter analyzer (HP4156C, Aglient, Santa Clara, CA, USA), followed by patterning the surface of the films with interdigital transducer (IDT) Al electrodes.

## 3. Results and Discussion

**Figure 1:**
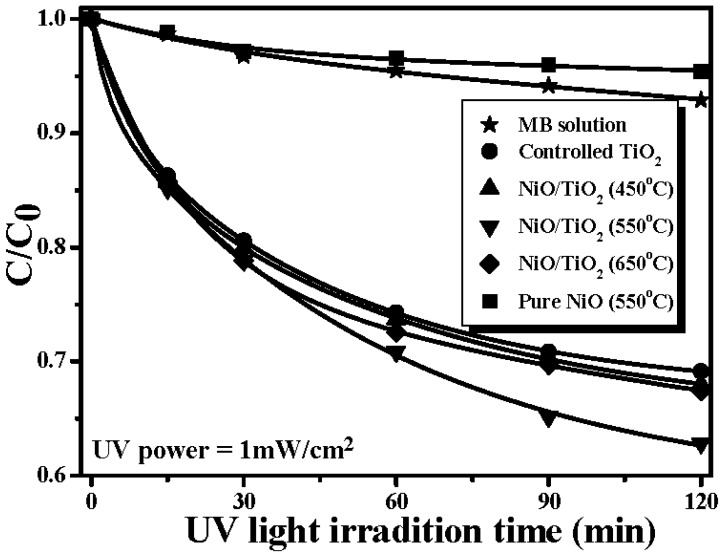
UV light-induced catalyses of the controlled TiO_2_ film, the NiO/TiO_2_ nanocomposite structures treated by the RTA (rapid thermal annealing) process at temperatures of 450 °C, 550 °C, and 650 °C, respectively, and the pure NiO film treated by the RTA process at 550 °C to degrade the MB (methylene blue) solution (the degradation of the MB solution directly irradiated by the UV light is provided for comparison).

**Figure 2:**
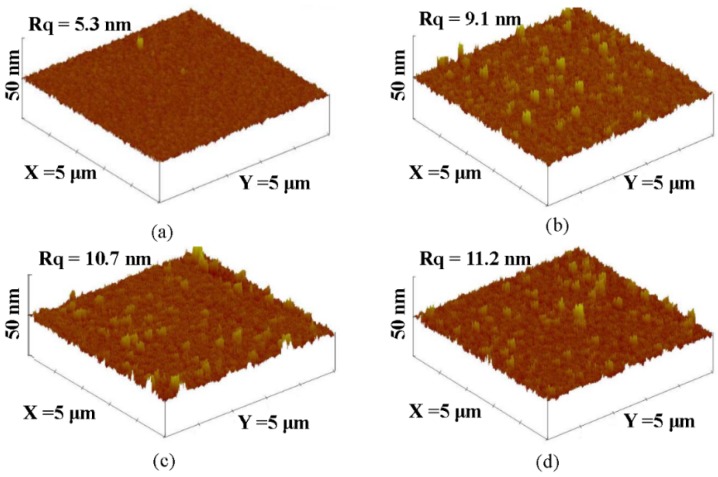
Surface roughness of the (**a**) controlled TiO_2_ film; and the NiO/TiO_2_ nanocomposite structures with an additive RTA process at temperature of (**b**) 450 °C; (**c**) 550 °C; (**d**) 650 °C, respectively.

**Figure 3:**
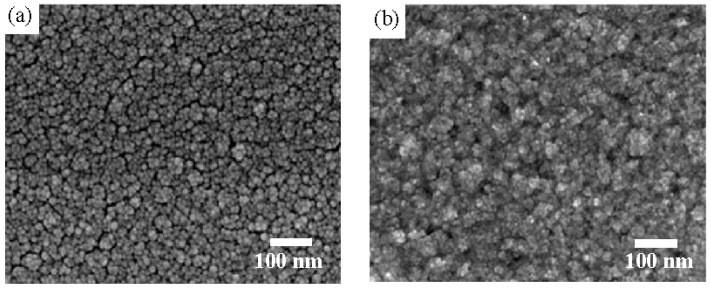
Surface morphologies of the (**a**) controlled TiO_2_ film and (**b**) the NiO/TiO_2_ nanocomposite structures annealed at 550 °C.

**Figure 4:**
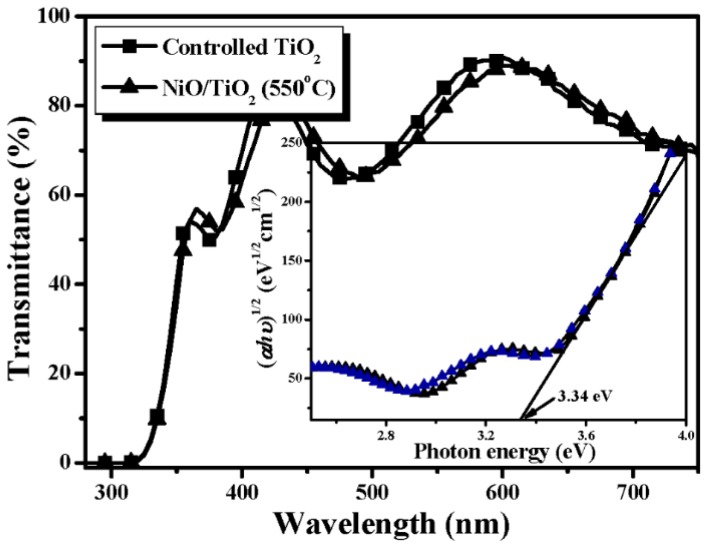
Optical transmittance of the controlled TiO_2_ film and the NiO/TiO_2_ nanocomposite structure annealed at 550 °C (the associated optical energy bandgaps also are derived from the inset figure).

**Figure 5:**
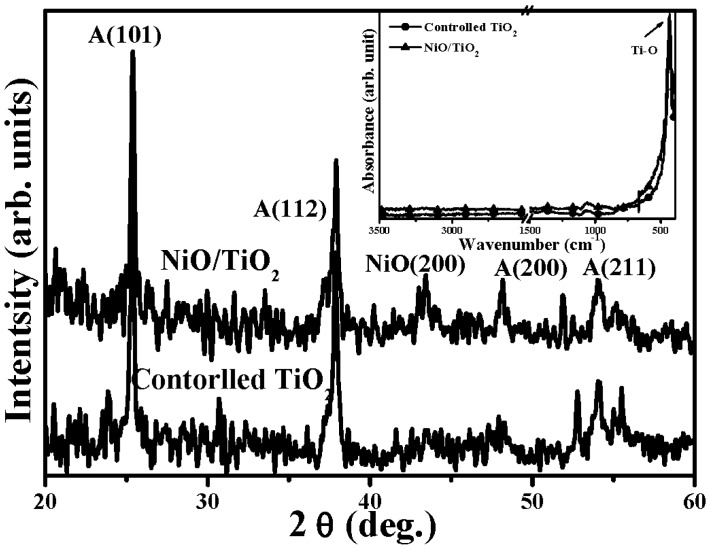
XRD patterns of the controlled TiO_2_ film and the 550 °C-annealed NiO/TiO_2_ nanocomposite structure (the associated FTIR spectra also are given in the inset figure).

**Figure 6:**
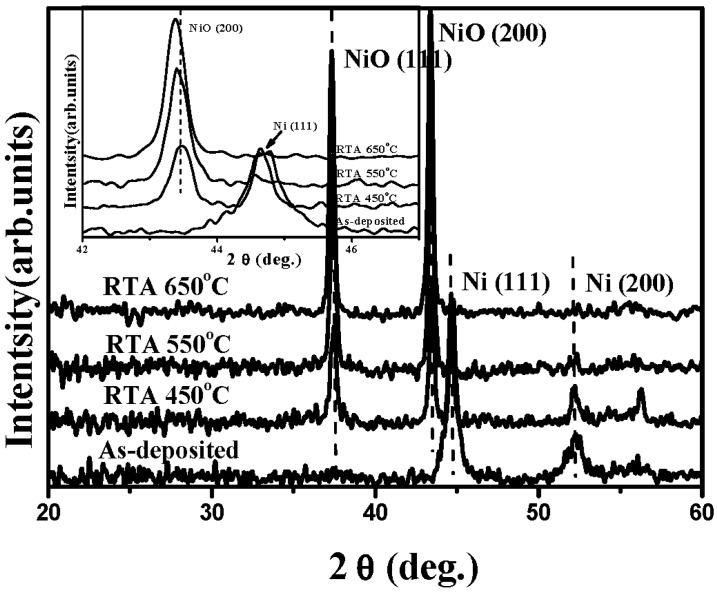
XRD patterns of the as-deposited Ni film and the films annealed at temperatures of 450 °C, 550 °C, and 650 °C, respectively (the inset figure shows a high resolution of the NiO(200) and Ni(111) planes).

Figure 1 shows the UV light-induced catalyses of the controlled TiO_2_ film, the NiO/TiO_2_ nanocomposite structures treated by the RTA process at temperatures of 450 °C, 550 °C, and 650 °C, respectively, and the pure NiO film treated by the RTA process at 550 °C to degrade the MB solution (the degradation of the MB solution directly irradiated by the UV light is provided for comparison). The UV light-induced photodegradation of the MB solution was significantly enhanced by introducing the controlled TiO_2_ film, while that of the pure NiO film was useless to degrade the MB solution. However, although the pure NiO film surface was inactive to the UV light irradiation, the NiO/TiO_2_ nanocomposite structure exhibited a faster photodegradation rate to the MB solution compared to that of the controlled TiO_2_ film. The rate constant, *k*, which represents the film’s photocatalytic activity, is evaluated from the photodegradation rate of the MB solution using the following equation [29]:

(1) $$\ln\left(\frac{c}{c_{0}}\right)=kt$$

where, *C* and *C_0_* are the concentration of the MB solution at UV light irradiation time of *t = 0* and *t*, respectively. Table 1 summarizes the rate constant (*k*) and root-mean-square surface roughness (*R_q_*) of the controlled TiO_2_ film and the NiO/TiO_2_ nanocomposite structure treated by the RTA process at temperatures of 450 °C, 550 °C and 650 °C, respectively. Clearly, all these TiO_2_ films with the surface nanocomposite structure possessed a higher rate constant to photo-degrade the MB solution than that of the controlled TiO_2_ film (0.0232 min^−1^). The best photocatalytic activity that corresponded to a rate constant of 0.0304 min^−1^ was obtainable from the Ni film deposited onto the TiO_2_ film surface and then rapidly annealed at 550 °C for 1 min under oxygen ambient. The surface roughness of the controlled TiO_2_ film and the NiO/TiO_2_ nanocomposite structures annealed at 450 °C, 550 °C and 650 °C, respectively, are illustrated in Figure 2a–d. As shown in Figure 2b–d, many significant protrusions were distributed over the RTA-treated NiO/TiO_2_ nanocomposite surface, thereby resulting in the obvious increase in the surface roughness. However, despite the fact that the surface roughness of the NiO/TiO_2_ nanocomposite structure annealed at 450 °C exhibited a high value of 9.1 nm compared to the controlled TiO_2_ film (~5.3 nm), which might be beneficial for enriching the surface specific area of the TiO_2_ film, the increase in the rate constant (~0.0236 min^−1^), as listed in Table 1, was limited compared to the controlled TiO_2_ film. In contrast to the 450 °C-annealed sample, the NiO/TiO_2_ nanocomposite structure annealed at 550 °C, which showed a surface roughness of 10.7 nm, possessed the highest rate constant of 0.0304 min^−1^. Figure 3a,b respectively, show the surface morphologies of the controlled TiO_2_ film and 550 °C-annealed NiO/TiO_2_ nanocomposite structures. Many fine grains were distributed over the controlled TiO_2_ film surface, whereas the grains were not apparent when the TiO_2_ film surface was coated with the thin NiO layer, and the surface was a feature of significant clusters which could be linked to the protrusions, as observed in Figure 2d. The possible reason for such surface morphology was attributed to the 20 nm-thick Ni layer grown in form of separate islands and then agglomerated by the sequential rapid oxidation process [30,31]. In addition, since the pure NiO film was inactive to the UV light irradiation, the increase in the surface roughness of the NiO/TiO_2_ nanocomposite structures as evidence of the NiO agglomeration would not have led to the enhancement of the photocatalytic activity. Accordingly, the rough surface measured from these annealed NiO/TiO_2_ nanocomposite structure was not the dominant mechanism responsible for the improvement in their rate constants, as listed in Table 1. Figure 4 depicts the optical transmittance of the controlled TiO_2_ film and the NiO/TiO_2_ nanocomposite structure annealed at 550 °C. It can be seen that both samples showed optical transparency within the visible wavelengths and then sharply decreased at the near UV wavelength. The average transmittance in the visible wavelengths (400–700 nm) of the annealed NiO/TiO_2_ nanocomposite structure (~77.5%) was only a little lower than that of the controlled TiO_2_ film (78.2%). In addition, the optical energy bandgap of the annealed NiO/TiO_2_ nanocomposite structure determined from the (*αhν*)^1/2^ versus the photon energy as shown in the inset figure, was identical to that of the controlled TiO_2_ film (~3.34 eV), indicating that the upper NiO film did not affect the optical absorption of the TiO_2_ film. The crystalline structures of the controlled TiO_2_ film and the 550 °C-annealed NiO/TiO_2_ nanocomposite structure are shown in Figure 5 (the associated FTIR spectra also are given in the inset figure). Though the signal was a little weak due to the total thickness of these films, only about 200 nm, the peaks showed evidence that the crystalline TiO_2_ still could be identified. The controlled TiO_2_ film exhibited a polycrystalline structure with only the anatase structure being dominated by the (101) phase determined from JCPDS 21-1272 (the diffraction peaks for the anatase phase are marked as “A”, with the diffraction planes given in parentheses). For the NiO/TiO_2_ nanocomposite system annealed at a temperature of 550 °C, the diffraction peaks and their positions were identical to those observed from the controlled TiO_2_ film, except for the appearance of the NiO (200) phase. The crystal size (D) of the two samples derived from the full width at half maximum (FWHM) of the preferred orientation of the A(101) phase adopting the Scherrer formula is expressed below [32]:

(2) $$D=\frac{k\lambda }{\beta \cos\theta }$$

where *k* (=0.9) is a constant, *λ* is the wavelength of the X-ray radiation (~0.154 nm), *β* is the FWHM in radians, and *θ* is the Bragg diffraction angle. The anatase crystallite size of the NiO/TiO_2_ nanocomposite structure estimated from the FWHM of the A(101) was about 27.3 nm, a value slightly smaller than that of the controlled TiO_2_ film (~28.9 nm). In addition, only one intense and sharp peak located at about 432 cm^−1^ was observed from both the FTIR spectra of these two samples, which was assigned as the Ti-O chemical bond [33], indicating that the deposition and the sequential RTA process did not result in a change to the crystalline structure or the bond states of the TiO_2_ film. In order to elucidate the mechanism responsible for the apparent increase in the photocatalytic activity of the NiO/TiO_2_ nanocomposite structure annealed at 550 °C, a thick Ni film was directly deposited on to the silicon substrate and then treated with the same RTA process. Figure 6 shows the XRD patterns of the as-deposited Ni film and the films annealed at temperatures of 450 °C, 550 °C, and 650 °C, respectively. The XRD pattern of the as-deposited Ni film included a significant peak at approximately 44.8° and a weak peak at 52.2°, which were in turn assigned as the Ni(111) and Ni(200) phase according to JCPDS 45-1027. When the Ni film was treated by the RTA process at a temperature of 450 °C, the diffraction peaks located at about 37.7° and 43.3°, which were classified as NiO(111) and NiO(200) according to JCPDS 47-1049, became the dominant signals in the XRD pattern, indicating crystalline evolution from the metallic Ni to the oxidized NiO structure. For the RTA temperatures of 550 °C and 650 °C, only two intense and sharp diffraction peaks identified as the NiO(111) and NiO(200) phases were obtained in their XRD patterns as evidence of the complete oxidation of the Ni film and the growth of the NiO crystallite structure. In addition, the shift of the NiO(200) diffraction peak toward a lower 2θ angle for the samples annealed at 550 °C and 650 °C shown in the inset figure was demonstrated as lattice expansion induced by interstitial oxygen atoms, which is consistent with previous research [34]. The electrical properties of the as-deposited Ni film, the films annealed at temperatures of 450 °C, 550 °C and 650 °C, respectively, and the controlled TiO_2_ film are listed in Table 2. The as-deposited Ni film performed its metallic nature with very high electron carriers of 8.23 × 10^23^ cm^−3^ and an ultra low resistivity (1.64 × 10^−6^ Ω·cm). However, as the metallic Ni and oxidized NiO structure coexisted in the sample annealed at a temperature of 450 °C, as shown in Figure 6, such phase separation resulted in the film’s resistivity being too large (higher than 10^5^ Ω·cm) to be measured. Notably, a high hole carrier of 2.60 × 10^18^ cm^−3^ was obtained from the metallic Ni completely transformed to the NiO structure as the film annealed at a temperature of 550 °C. The activation of the interstitial oxygen atoms that were classified as acceptors in the annealed NiO film, was one possible reason for the conversion of the conduction from *n*-type to *p*-type behavior. Moreover, as an elevated annealing temperature on the film was also prone to cause outdiffusion of the atoms from the film surface, especially for the oxygen atoms, which acted as donor defects (*i.e.*, oxygen vacancies) [35], the hole concentration of the NiO film annealed at 650 °C was thus apparently decreased to 1.29 × 10^14^ cm^−3^ as a consequence of the compensation of the hole carriers. These NiO films, which showed p-type conduction in contact with the controlled TiO_2_ film, that performed n-type with a concentration of 3.55 × 10^13^ cm^−3^, as listed in Table 2, thus achieved the surface *p-n* heterojunction structure.

**Table 1:** Rate constant (*k*) and root-mean-square surface roughness (*R_q_*) of the controlled TiO_2_ film and the NiO/TiO_2_ nanocomposite structure treated by the RTA process at temperatures of 450 °C, 550 °C, and 650 °C, respectively.

Values	Controlled TiO_2_	NiO/TiO_2_ (450 °C)	NiO/TiO_2_ (550 °C)	NiO/TiO_2_ (650 °C)
*k* (min^−1^)	0.0232	0.0236	0.0304	0.0251
*Rq* (nm)	5.3	9.1	10.7	11.2

**Table 2:** Electrical properties of the as-deposited Ni film, the controlled TiO_2_ film, and the films annealed at temperatures of 450 °C, 550 °C and 650 °C, respectively.

Samples	Concentration (cm^−3^)	Mobility (cm^2^·V^−1^·s^−1^)	Resistivity (Ω·cm)
As-deposited Ni	-8.23 × 10^23^	4.74	1.64 × 10^−6^
NiO (450 °C)	N/A	N/A	N/A
NiO (550 °C)	2.60 × 10^18^	21.6	1.11 × 10^−1^
NiO (650 °C)	1.29 × 10^14^	4.30	1.13 × 10^4^
Controlled TiO_2_	-3.55 × 10^13^	40.8	4.32 × 10^3^

**Figure 7:**
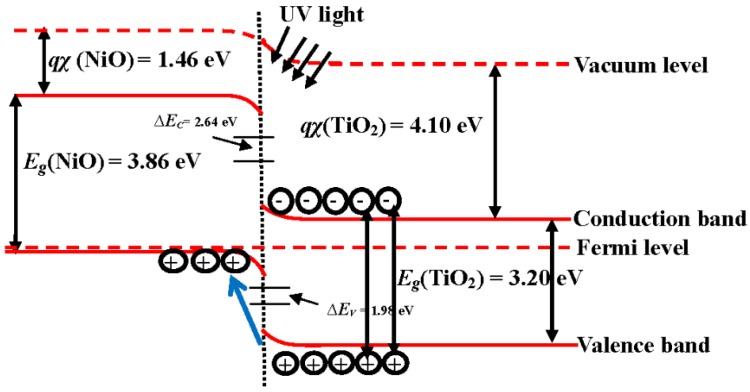
A schematic energy band diagram of the 550 °C annealed *p*-NiO/*n*-TiO_2_ heterojunction structure.

**Figure 8:**
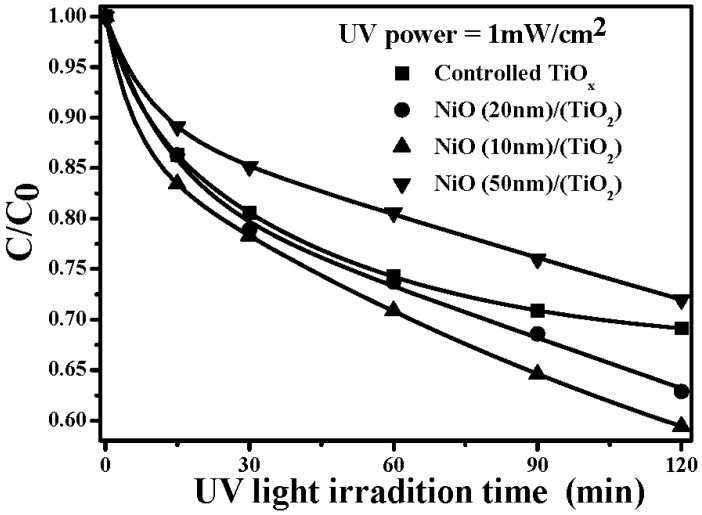
UV light-induced catalysts of the MB solution using the 550 °C-annealed *p*-NiO/*n*-TiO_2_ nanocomposite structures, with the thickness of the NiO film being 10, 20 and 50 nm, respectively.

**Figure 9:**
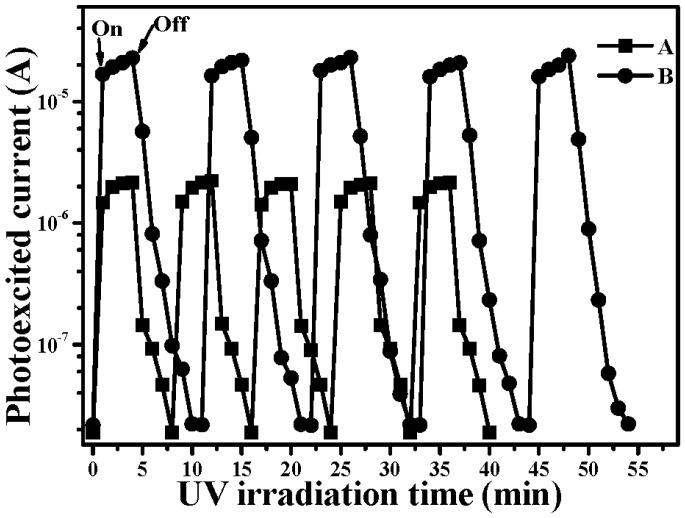
Transient photo-excited current responses of the controlled TiO_2_ film (curve A) and the 550 °C-annealed NiO/TiO_2_ nanocomposite structure (curve B).

A schematic energy band diagram of the 550 °C annealed *p*-NiO/*n*-TiO_2_ heterojunction structure adopting Anderson’s rule is plotted in Figure 7. The bandgaps, *Eg*, of NiO and TiO_2_, which have been taken from the reported works, are 3.86 and 3.20 eV, respectively, while the electron affinities, *qχ*, for these two materials are 1.46 and 4.10 eV, respectively [36,37,38,39]. As can be seen in this figure, the band offset for holes (1.98 eV) is a little smaller than that for electrons (2.64 eV). In addition, a space charge region originating from the *p-n* junction led to an inner electrical field in the NiO/TiO_2_ interface. Thus, the photo-generated electron-hole pairs in the TiO_2_ film surface under UV light irradiation would be effectively separated with the effect of the built-in voltage formed by the *p*-NiO/*n*-TiO_2_ heterojunction. The photon-generated holes in the TiO_2_ film surface could easily flow into the NiO film, whereas the photo-generated electrons were blocked by the significant barrier and restrained in the TiO_2_ film surface. The life time of these photo-generated carriers in the *p*-NiO/*n*-TiO_2_ nanocomposite structure was thus expected to be prolonged as a consequence of the inhibition of their recombination. Accordingly, an apparent increase in the rate constant evaluated from the decomposition to the MB solution, as listed in Table 1, was obtainable from the NiO/TiO_2_ nanocomposite structure with an RTA treatment at a temperature of 550 °C. The UV light-induced catalysts for the MB solution using the 550 °C-annealed *p*-NiO/*n*-TiO_2_ nanocomposite structures, with the thickness of the NiO film being 10, 20 and 50 nm, respectively, are illustrated in Figure 8. It is worth noting that, although the *p*-NiO/*n*-TiO_2_ heterojunction structure was demonstrated to be beneficial for separating the photo-generated electron-hole pairs, the thickness of the NiO film also strongly affected the resulting photocatalytic activity of the MB solution. The photodegradation rate of the MB solution was further increased for the NiO/TiO_2_ nanocomposite structure with a NiO film thickness of 10 nm (*k* ~ 0.0327 min^−1^), whereas the 50 nm-thick NiO film in contact with the TiO_2_ film surface, resulted in an apparent reduction in the rate constant (0.0144 min^−1^), as evaluated from the photo-induced decomposition to the MB solution. This revealed that a thin NiO film that corresponded to a short diffusion path for the photo-induced holes which migrated to the structural surface to react with the MB molecules was also crucial for affecting the resulting photocatalytic activity. Figure 9 shows the transient photo-excited current responses of the controlled TiO_2_ film and the 550 °C-annealed NiO/TiO_2_ nanocomposite structure. The photo-induced catalysts of both samples were reliable as evidence of the repeatable photocurrent responses. The dark current of the NiO/TiO_2_ nanocomposite structure (~2.2 × 10^−8^ A) was only a little higher than that of the controlled TiO_2_ film (~1.9 × 10^−8^ A) under the same irradiation power, whereas its photo-induced current (~2.3 × 10^−5^ A) was an order of magnitude higher than that of the controlled TiO_2_ film (~2.2 × 10^−6^ A), revealing the increase in the quantum efficiency accomplished by this *p-n* heterojunction structure. The recombination time of the photo-generated electro-hole pairs evaluated from the on-off process was also prolonged from 4 min to 7 min as the 10 nm-thick Ni film was deposited onto the controlled TiO_2_ film and rapidly oxidized at 550 °C for 1 min under oxygen ambient.

## 4. Conclusions

Thin Ni metal was deposited onto TiO_2_ film and rapidly oxidized at 450 °C, 550 °C and 650 °C, respectively, for 1 min under oxygen ambient to result in a surface with the NiO/TiO_2_ nanocomposite structure. Although the RTA process on the NiO/TiO_2_ nanocomposite structure did not result in a change to the crystalline structure and optical absorption of the TiO_2_ film and the surface NiO film were also inactive in response to UV light irradiation, these samples possessed a higher rate constant than that of the controlled TiO_2_ film, as evaluated from the films decomposing the MB solution. According to the investigations of the crystalline structure and the electrical properties of the single NiO films, the evolution of the photocatalytic activities of these annealed NiO/TiO_2_ nanocomposite structures were strongly correlated to the conduction type and carrier concentration of the NiO films. The 450 °C-annealed NiO/TiO_2_ nanocomposite structure, of which the NiO film showed insulated behavior due to the phase separations of Ni and NiO, possessed a rate constant (0.0236 min^−1^) only slightly higher than that of the controlled TiO_2_ film (0.0232 min^−1^). By contrast, the rate constant of the 550 °C-annealed NiO/TiO_2_ nanocomposite structure, in which the NiO film behaved as *p*-type conduction with a high carrier concentration of 2.60 × 10^18^ cm^−3^, was apparently increased to 0.0304 min^−1^. In addition, the rate constant was again decreased to 0.0251 min^−1^ for the sample annealed at 650 °C while the NiO film had a low hole concentration of 1.29 × 10^14^ cm^−3^. The reason responsible for the improvement in the photocatalytic activity was the formation of the surface *p-n* heterojunction achieved from the annealed NiO/TiO_2_ nanocomposite structure. The inner electrical field that appeared at the *p*-NiO/*n*-TiO_2_ interface was favorable for the separation of the photo-generated electron-hole pairs in the TiO_2_ film surface, thereby improving the quantum efficiency as demonstrated from the increase in the photocurrent. The recombination of the photo-generated carriers determined from the on-off time of the photocurrent was also apparently prolonged to 7 min compared to that of the controlled TiO_2_ film. In addition, by reducing the thickness of the NiO film to 10 nm, the photocatalytic activity of the resulting NiO/TiO_2_ nanocomposite structure was further enhanced with a rate constant of 0.0327 min^−1^ as a consequence of the photo-induced carriers, which could migrate to the structural surface more efficiently.
